# Characterization of 13 multi-drug resistant *Salmonella *serovars from different broiler chickens associated with those of human isolates

**DOI:** 10.1186/1471-2180-10-86

**Published:** 2010-03-23

**Authors:** Lan-Ho Chiu, Cheng-Hsun Chiu, Yan-Ming Horn, Chien-Shun Chiou, Chien-Yu Lee, Chia-Ming Yeh, Chang-You Yu, Chean-Ping Wu, Chao-Chin Chang, Chishih Chu

**Affiliations:** 1 Animal Disease Control Center Chiayi County, Taibao 1st Rd., Taibao City, Chiayi County 612, Taiwan; 2 Department of Pediatrics, Chang Gung Children's Hospital, 5 Fu-Hsing Street, Kuei-Shan Hsiang, 33375, Taoyuan, Taiwan; 3 Department of Medicine, College of Medicine, Chang Gung University, 259 Wen-Hwa 1st Road, Kwei-Shan, Taoyuan, 33302, Taiwan; 4 Department of Animal Science, National Chiayi University, No 300, University Rd, Chiayi, 60004, Taiwan; 5 The Central Region Laboratory, Center of Research and Diagnostics, Centers for Disease Control, No 30, Wenxin S 3rd Rd, Nantun Dist, Taichung, 40856, Taiwan; 6 Department of Veterinary Medicine, National Chiayi University, No 300, University Rd, Chiayi, 60004, Taiwan; 7 Graduate Institute of Veterinary Public Health, School of Veterinary Medicine, National Chung Hsing University, 250, Kuo Kuang Road, Taichung 40200, Taiwan; 8 Department of Microbiology and Immunology, National Chiayi University, No 300, University Rd, Chiayi, 60004, Taiwan

## Abstract

**Background:**

*Salmonella *are frequently isolated from chickens and their products. Prevalent serogroups and serovars of *Salmonella *as well as their genotypes and antibiograms were determined for cloacal samples from 1595 chickens. To understand the possible serovar and H antigens for transmission between chicken and human, serovars and their H antigens of 164 chicken and 5314 human isolates were compared.

**Results:**

Prevalence of *Salmonella *differed among chicken lines and ages. Chicken and human isolates belonged mainly to serogroup B, C1, C2-C3, D, and E. 13 serovars and 66 serovars were identified for chicken and human isolates respectively. The common serovars for chicken and human isolates were *S*. Typhimurium, *S*. Enteritidis, *S*. Albany, *S*. Derby, and *S*. Anatum and shared common H1 antigens "g complex; i; e,h; and z4,z24" and H2 antigens "1 complex and -". In human isolates, H1 antigen "i" and H2 antigen "-" were common in all serogroups. In chicken, antimicrobial susceptibility differed among serogroups, serovars and three counties. All isolates were susceptible to cefazolin and ceftriaxone, but highly resistant to ampicillin, chloramphenicol, flumequine, streptomycin, sulfamethoxazole-trimethoprim, and tetracycline. Except those isolates of serogroup C1 of Chick group and serogroup G, all isolates were multi-drug resistance. Only *S*. Kubacha, *S*. Typhimurium, *S*. Grampian, and *S*. Mons were resistant to ciprofloxacin and/or enrofloxacin.

**Conclusion:**

In chicken, prevalent serogroups and serovars were associated with chicken ages, lines and regions; and flouroquinolone-resistant and MDR isolates emerged. H1 antigens "g complex and i" and H2 antigens "1 complex and -" might be important for transmission of *Salmonella *between chicken and human.

## Background

*S*. Enteritidis and *S*. Typhimurium, as two main zoonotic and broad-host-range pathogens that cause human salmonellosis, have been frequently isolated from poultry and their products [1-8]. Prevalence of *Salmonella *differs between layers and broilers [9,10]. Factors influencing the prevalence of chicken-associated *Salmonella *are feeds and growth environment [11], transportation process [12,13], and chick sources [14]. Moreover, age-associated prevalence has been reported in layers, maximal prevalence at 18 weeks before egg production and gradually decreases with aging [15]. In broiler the prevalence differed depending on sale sites from 17.9% in slaughterhouses [16] and up to nearly 100% in the open markets and supermarkets [17].

Appearance of monophasic variants such as in *S*. Typhimurium [4,5,12:1:-] [18,19] increases the problem in serotyping. Therefore, molecular methods have been developed to differentiate the serovars based on the nucleotide sequence variations in flagellar structural genes *fliC *and *fljB *[20-22] and PFGE analysis [15,23,24]. Prevalent serovars differ between chickens and ducks [25] and are associated with chicken lines and geographic area [15,25-27]. In Taiwan, we reported that *Salmonella *serogroup C1 and B, especially *S*. Typhimurium, were predominant *Salmonella *in duck and geese [7,8]. In another study of duck, the prevalence of *Salmonella *was 4.6% and *S*. Potsdam, *S*. Dusseldorf, and *S*. Indiana were the predominant serovars [28]. Therefore, we analyzed the prevalence of *Salmonellae *among different chicken sources and determined serotypes by PFGE analysis first, followed by traditional agglutination test of each genotype. After characterizing antibiograms and genomic variations in chromosome and plasmid of chicken isolates, flagellar antigens of chicken and human isolates were compared to understand the common antigens possibly for transmission of *Salmonella *between human and chicken.

## Methods

### Sample collection and enrichment

Totally 1595 chickens of 1-year-old broiler breeder, 1-day-old chicks (Chick) and 9-week-old chickens (NHC) of Taiwan broiler chicken, 1-year-old layers and 3-week-old broiler were sampled by 108C Amies Agar Gel - Single plastic swab (Copan Diagnostic Inc. Murrieta CA 92562 USA) from cloaca of each chicken fed at different farms in Chiayi of Taiwan from 2002 to 2003. Layers and broilers were fed in commercial cage and house farm respectively. The sampled swabs were grown in 9 mL of gram-negative broth (GN, Difco 0486) at 37°C for 24 h. Over-night GN bacterial broth was streaked on xylose lysine deoxycholate (XLD, Difco 0788) plates, which were incubated at 37°C for 24 h. Black colonies were further examined by biochemical tests including triple sugar iron agar (TSI), Christensen's urea agar (URE), Simmons' citrate agar (CIT), sulfide-indole-motility medium (SIM), Voges-Proskauer medium (VP), Moller's ornithine decarboxylase medium (ORN), lysine iron agar (LIA) and mobility-indole-ornithine agar (MIO) purchased from Merck (Taiwan). At least two positive isolates from each plate were maintained on brain heart infusion agar (BHIA). In addition, *Salmonellae *from 9-week-old NHC in Tainan (36 isolates) and Pintung (30 isolates) at same period were also analyzed.

### Serogroup and serotype identification

*Salmonella*-positive isolates were further serogrouped by the slide agglutination test with the use of O-antigen antiserum and serotyped by the tube agglutination test with the use of H-antigen antisera. Both antisera were purchased from Difco (Becton Dickinson Co., Franklin Lakes, NJ, USA). In addition, 5314 *Salmonellae *were collected from 19 medical centers and district hospitals located throughout the countries from 2003 to 2005 and serotyped in the *Salmonella *Reference Laboratory of Centers for Disease Control (CDC), Department of Health, Taiwan, with antisera purchased from S&A Reagents Lab (Bangkok, Thailand), Denka Seiken (Tokyo, Japan), Statens Serum Institut (Copenhagen, Denmark), and a local biotech company, LTK Biolaboratories (Taoyuan, Taiwan). Phase induction was performed using a paper-bridged method developed in the laboratory of Taiwan CDC [29].

### Antimicrobial susceptibility test

Each isolate was examined by disk diffusion method for its susceptibility to the antimicrobial agents including ampicillin (A, 10 μg), cefazolin (CZ, 30 μg), ceftriaxone (Cro, 30 μg), chloramphenicol (C. 30 μg), streptomycin (S, 10 μg), sulfamethoxazole-trimethoprium (Sxt, 1.25/23.75 μg), and tetracycline (T, 30 μg). In addition, resistance to three fluoroquinolones: flumequine (Ub, 30 μg) of limited spectrum quinolone and enrofloxacin (En, 5 μg) as well as ciprofloxacin (Ci, 5 μg) of broad spectrum quinolone. While single bacterial colony was taken into 5 ml of Mueller-Hinton broth (MHB; Merck, Taiwan) and cultured at 37°C for 8 hrs, bacterial broth was then adjusted to 0.5 Mcfarland and plated on Mueller-Hinton agar (MHA; Merck, Taiwan). Antimicrobial disks (BD Diagnostic systems, USA) were plated onto MHA agar and then incubated at 37°C for 18 hrs. Susceptibility and resistance were determined according to the interpretation criteria to *E. coli *(ATCC No. 25922) established by Clinical Laboratory Standards Institute (CLSI) standard [30]. Multi-drug resistance (MDR) isolate is defined as that isolate resistance to two or more antibiotics belonging to different antibiotic classes.

### Plasmid and genotype analysis

Plasmid DNA pattern was determined by Kado and Liu method [31] and purified plasmid DNA was subjected to gel electrophoresis with 0.6% SeaKem GTG agarose (Cambrex Bio Science Rockland, Inc, Rockland, ME, USA) at 50 V for 2.5 hrs. Genotypes of all isolates were determined by PFGE analysis with restriction endonuclease *Xba*I digestion. The procedure of PFGE analysis was described earlier [32]. The digested DNA was separated by CHEF Mapper XA system (BioRad, Hercules, California, USA) in 0.5 × TBE at 14°C for 22 h with Auto-Algorithm model of 30-600 kb, 6 V/cm, switching interval 4.0-70.0 sec. The genotypes were defined as 3 band differences between two isolates [33].

## Results

### Prevalent serogroups and serovars among chicken lines and locations

Prevalence of *Salmonella *differed between chicken lines (0% for layer vs 0.3% for breeder broiler and 11.3% for broiler) and ages from 10.3% for Chick and 3.8% for NHC of Taiwan broiler chicken (Table 1). 164 *Salmonella *isolates belonged to serogroup C1, B, D, C2-C3, E, and G in the decreasing order and the number of serogroups differed among 3 counties. Further, region-specific serogroups were identified as serogroup G in Chiayi, serogroup D in Tainan, and serogroup C2-C3 and E in Pintung (Table 1). In Chiayi, age-associated serogroups were found for serogroup C1 *Salmonella *in Chick group and serogroup B and G in NHC group (Table 1).

**Table 1:** Prevalence of *Salmonella *serogroups in different layer- and broiler chickens in three Counties

	**County**^a^								
		
Serogroup	Chiayi						Tainan	Pintung	Total isolates
				
	Layer	Breeder	Broiler	NHC^b^	Chick^c^	Total	NHC	NHC	
B	0	1	16	2	0	19	13	7	39
C1	0	0	1	0	77	78	2	8	88
C2	0	0	0	0	0	0	0	11	11
D	0	0	0	0	0	0	18	0	18
E	0	0	0	0	0	0	0	5	5
G	0	0	0	3	0	3	0	0	3
Total	0	1	17	5	77	99	33	31	164
Prevalence	0	0.3	11.3	3.8	10.3	6.2			
(%)	(0/285)	(1/280)	(17/150)	(5/130)	(77/750)	(99/1595)			

^a ^The number of each serogroup was determined in our laboratory by examination of *Salmonella *isolated from cloacal samples of chicken in Chiayi County and from surveillance of Tainan and Pintung County.

^b^ NHC: 9-wk-old Native Hybrid Chickens (simulated native chicken) of Taiwan broiler chickens

^c^ Chick: one-day-old NHC chicks.

**Figure 1:**
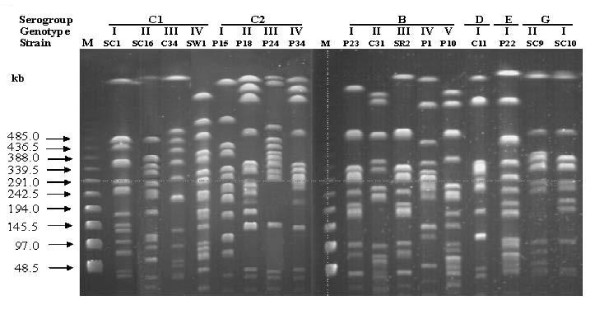
**XbaI-digested PFGE genotypes of each Salmonella serogroups**. M: lamda ladder size marker. SC1: non-typable serogroup C1 *Salmonella*. SC16: *S*. Redba. C34: *S*. Derby. SW1: *S*.Grampian. P15: *S*. Blockley. P18, P24, and P34: *S*. Albany. P23: *S*. Mons. C31: *S*. Typhimurium var. Copenhagen. SR2: *S*. Kubacha. P1: *S*. Derby. P10: *S*. Typhimurium. C11: *S*. Enteritidis. P22: *S*. Anatum. SC9 and SC10: *S*. Havana. Genotypes I to IV are defined as difference more than 3 bands between two isolates [33].

164 *Salmonella *isolates were firstly examined for their genotypes by *Xba*I-PFGE analysis (Figure 1) and further isolates of each genotype were serotyped by traditional agglutination method. In total, 18 PFGE patterns belonged to 13 serovars (Table 2). Except *S*. Albany and *S*. Havana that consisted of multiple genotypes, PFGE genotypes matched exactly with serotypes. 13 serovars were *S*. Derby, *S*. Kubacha, *S*. Mons, and *S*. Typhimurium (containing *S*. Typhimurium var. Copenhagen) of serogroup B, *S*. Choleraesuis (containing non-typable serovar), *S*. Grampian, *S*. Hissar, and *S*. Redba of serogroup C1, *S*. Albany and *S*. Blockley of serogroup C2-C3, *S*. Enteritidis of serogroup D, *S*. Anatum of serogroup E and *S*. Havana of serogroup G (Table 2). Predominant serovar in each serogroup was *S*. Mons, not *S*. Typhimurium, in serogroup B, *S*. Choleraesuis from Chick and *S*. Grampian from NHC in serogroup C1, and *S*. Albany in serogroup C2-C3 (Table 2).

**Table 2:** Characterization of *Salmonella *isolates by 4 methods

Serogroup	Serovar	County	Chicken lines	Resistance type^a^	PFGE genotype^b^	Plasmid type^c^	Total isolates
	Derby	Pintung	NHC	E	IV	5	1
	
		Pintung	NHC	M	IIIa	2a	2
	Kubacha						
		Chiayi	NHC Broiler	J	IIIa	4a 1	1 1
	
			Broiler	I J	I	1	12 3
		Chiayi	NHC	K	I d	1a	1
			Breeder	C	I e	2b	1
		
		Pintung	NHC	G	I	1b	1
		
B	Mons				I	2	4
						1b	2
				J	I a	1a	2
		Tainan	NHC		I	3	1
						1d	1
						1c	1
				K	Ia	1b	1
	
	Typhimurium var. Copenhagen	Tainan	NHC	L	II	4	1 1
	
	Typhimurium	Pintung	NHC	M D	V	3a 6	2 1

	Choleraesuis	Chiayi	Chick	A	III IIIa IIIb	1 5	59 1 1
	
		Tainan		G		3	1
C1	Grampian		NHC		IV	1a	1
		Pintung		M		1	7
						1a	1
	
	Hissar	Chiayi	Broiler	I	V	4	1
	
	NT^d^	Chiayi	Chick	A	I	1 2	5 10
	
	Redba	Chiayi	Chick	A	II	5	1

	Blockley	Pintung	NHC	E	I	1	1
	
C2					II		3
	Albany	Pintung	NHC	J	III	1	5
					IV		2

				F		2	7
D	Enteritidis	Tainan	NHC		I	3	3
						1	7
				B		2	1

E	Anatum	Pintung	NHC	J H	I	1 2	3 1

G	Havana	Chiayi	NHC	A	I II	1	2 1

^a^Antibiogram of each isolate was determined by the resistance to antimicrobials ampicillin (A), chloramphenicol (C), ciprofloxacin (Ci), ceftriaxone (Cr), cefazolin (Cz), enrofloxacin (En), flumequine (Ub), streptomycin (S), sulfamethoxazole-trimethopriem (Sxt), tetracycline (T). The association of resistance type with antibiogram was the followings: resistance type A for antibiogram S, B for Ub, C for UbS, D for ST, E for SxtUb, F for CSTUb, G for ASSxtTUb, H for ACSSxtT, I for CSSxtTUb, J for ACSSxtTUb, K for ACEnSSxtTUb, L for ACCiEnSxtTUb, and M for ACCiEnSSxtTUb.

^b^PFGE genotypes was determined by 3 band differences between two isolates [Figure 1, [32]].

^c^Plasmid was analyzed by Kado and Liu method (30, supplementary Figure 1). Plasmid profile was determined by plasmid size and number (supplementary Table 2).

^d^NT: non-typable

### Antimicrobial susceptibility

All isolates were susceptible to CZ and Cro. In contrast to resistance only to streptomycin for 77 *S*. Choleraesuis isolates in Chick group and two isolates of serogroup G, all isolates were MDR (Table 3). Serogroup B, C2-C3 and E were highly resistance to A, C, S, Sxt, T and Ub. However, serogroup D was relatively low in resistance to above antimicrobials. Serogroup and serovars isolated from broiler and NHC group differed in resistance to three quinolone antimicrobials. Except serogroups E and G, all serogroups, were nearly 100% resistance to Ub and only serogroups B and C1 were resistant to En and Ci (Table 3). Among 164 isolates, we only found 4 En-resistant *S*. Mons and 13 En and Ci-resistant isolates including 2 *S*. Kubacha isolates, 2 *S*. Typhimurium isolates, and 1 *S*. Typhimurium var. Copenhagen isolates of serogroup B and 8 *S*. Grampian isolates of serogroup C1 (Table 2). Importantly, near 40% of isolates from Pintaung were resistant to En and Ci. According to resistance to 9 antimicrobials tested, 13 antibiograms differed among serogroups and serovars (Table 2 and 3). Highest drug-resistant types L with antibiogram ACCiEnSxtTUb and M with antibiogram ACCiEnSSxtTUb were only found in serogroup B and C1 of NHC group from Pintung mostly and Tainan. *Salmonella *genomic island (SGI) related ACSSuT resistance was found in serogroup B, C2 and E. Resistance to antimicrobials tested varied among 3 counties (Table 3 and Additional file 1: Table S1). Highest resistance was found in isolates from Pintung, followed by Tainan, and Chiayi and lowest Sxt resistance rate was observed in isolates from Tainan.

**Table 3:** Differences in prevalence of resistance to 9 antimicrobials among serogroups and Counties

Antimicrobials^a^	Serogroup (%)						County (%%)		
		
	B	C1	C2	D	E	G	Chiayi	Tainan	Pintung
A	61.5	11.4	100	0	100	0	23.8	47.1	77.4
C	89.7	10.2	91	0	100	0	90.5	70.6	74.2
Ci	12.8	9.1	0	0	0	0	0	2.9	38.7
En	20.5	9.1	0	0	0	0	4.7	8.8	38.7
S	97.4	100	91	55.6	100	100	100	76.5	93.5
Sxt	94.9	12.5	91	0	100	0	85.7	47.1	96.8
T	94.9	12.5	91	55.6	100	0	85.7	76.5	93.5
Ub	97.4	12.5	91	100	60	0	90.5	100	90.3

^a ^A for ampicillin, C for chloramphenicol, Ci for ciprofloxacin, En for enrofloxacin, S for streptomycin, Sxt for sulfamethoxazole-trimethoprime, T for tetracycline, and Ub for Ub for flumequine.

### Plasmid profile analysis

Based on plasmid number and size determined by gel electrophoresis and plasmid size marker 50 kb and 90 kb of OU7526, in total 19 plasmid profiles were identified and the plasmid profiles and their number differed among serogroups and serovars (Additional file 2: Table S2; Additional file 3: Figure S1). Among 13 serovars, *S*. Albany, *S*. Blockley, *S*. Havana, and *S*. Redba as well as few isolates of *S*. Choleraesuis, *S*. Enteritidis, and *S*. Typhimurium lacked plasmid. All other serovars harbored at least one plasmid and differed in plasmid profile.

### Serovar association between chicken and human isolates

*S*. Albany, *S*. Anatum, *S*. Choleraesuis, *S*. Derby, *S*. Enteritidis, and *S*. Typhimurium were in common for 13 chicken serovars and 66 human serovars and other 7 serovars of chicken isolates were not or barely observed in human (Table 2, 4 and 5). Total serovar number of each serogroup decreased from serogroup C1, B, C2, E to D for human isolates (Table 4). Despite of the presence of 66 serovars, there were only presence of 11 H1 antigens including b, c, d, j, k, r, y, eh, g-complex, and z-complex and 5 H2 antigens including -, z_6_, lw, 1-complex, and en-complex (Table 4). Common antigens in all serogroups were "i" for H1 antigen: and "-" for H2 antigen. In compared the chicken and human isolates from Taiwan, United Kingdom and United States, the common serovars were *S*. Typhimurium, *S*. Enteritidis, *S*. Anatum, and *S*. Derby with common antigens of . "g complex; i; z_4_,z_24_; and e,h" for H1 antigen and "- and 1 complex" for H2 antigen (Table 5).

**Table 4:** The H1 and H2 antigens of 66 *Salmonella *serovars of human isolates collected from 2003 to 2005

	Serogroup			B	C1	C2	D	E	Others
		
H antigen				11	19	9	7	8	12
H1	b			±^a^	-	-	-	+	-
	c			-	+	-	-	-	-
	d			+	-	+	+	-	+
	i			+	+	+	+	+	+
	k			+	+	+	-	-	-
	r			-	+	-	-	+	-
	y			-	+	-	-	-	-
	e,h			-	-	-	-	+	-
	g complex								
	f,g/f,g,s/[f],g,m, [p]/g,p			+/+/-/-^b^	-/-/-/-	-/-/-/-	-/-/+/+	-/-/-/-	-/-/-/-
	g,m, [s]/g,m, [p],s/g,s,t			-/-/-	-/+/-	+/-/-	-/-/+	-/-/+	-/-/-
	l complex								
	l,v/l,w/l,z13			-/-/-	-/-/-	-/-/-	+/+/-	-/-/+	+/-/-
	z complex								
	z/z4/z10/z29/z38			+/-/+/-/-	+/-/+/+/-	-/+/+/-/-	-/-/-/-/-	-/-/-/-/-	-/+/-/-/+
	Total antigens			6	7	5	4	5	4

	-			+	+	+	+	+	+
	l,w			-	-	-	-	+	+
	z6			-	+	+	-	-	-
H2	1 complex								
	1,2/1.5/1,7/[1,2,7]			+/+/+/-	+/+/+/+	+/+/±/-	-/+/-/-	+/+/-/-	-/-/-/-
	en complex								
	e,n,x/e,n,z_15_			-/-	+/+	+/-	-/+	-/-	-/-
	Total antigens			2	4	4	3	3	2

^a ^± means presence (+) or absence (-) of b antigen.

^b ^+/+/-/- indicates presence (+) of antigens f,g/f,g,s and absence (-) of antigens [f],g,m, [p]/g,

**Table 5:** Serovars of chicken isolates associated with those of human isolates collected from 2003 to 2005

					Prevalence (%) of serovar of chicken and human isolates from different area											
		
			H antigen		2003				2004				2005			
			
Serovars of chicken isolates in this study					Chicken		Human		Chicken		Human		Chicken		Human	
					
			1	2	USA^a^	UK^b^	USA	T^c^	USA	UK	USA	T	USA	UK	USA	T
Serogroup B																
Derby			f,g	[1,2]	0.2	0.3	0.3	2.4	0	0	3.8	2.7	0.03	0.2	0.34	2.3
Kubacha			l,z_13_,z_28_	1,7	0	0	0	0	0	0	0	0	0	0	0	0
Mons			d	l,w	0	0	0	0	0	0	0	0	0	0	0	0
Typhimurinum			i	1,2,[7]	4.7	2.8	15.8	25.2	6.7	1.7	16.5	22.3	318	1.4	16.5	24.7
Serogroup C1																
Choleraesuis			c	1,5	0	0	0.03	4.2	0	0	0.05	4.3	0.03	0	0.02	2.0
Grampian			r	l,w	0	0	0	0	0	0	0	0	0	0	0	0
Hissar			c	1,2	0	0	0	0	0	0	0	0	0	0	0	0
Redba			z_10_	z_35_	0	0	0	0	0	0	0	0	0	0	0	0
Serogroup C2-C3																
Blockley			k	1,5	0	0	0.18	0	0	0	0.23	0	0.05	0	0.14	0
Albany			Z_4_,z_24_	-	0	0	0.05	4.7	0.6	0	0.09	3.4	0.03	0	0.10	4.9
Serogroup D1																
Enteritidis			[f],g.m. [p]	[1,7]	3.8	5.2	13.1	22.7	9.8	1.8	14.10	22.9	4.7	4.5	18.6	24.4
Serogroup E																
Anatum			e,h	1,6: [z_64_]	0.5	0.6	0.47	1.0	0	0	0.7	1.1	0.64	0.6	0.54	0.7
Serogroup G																
Havana			f,g, [s]	-	0.2	1.2	0.08	0	0.6	0.7	0.089	0.1	0.27	0.8	0.07	0
Total			*Salmonellae*		2038	924	37442	529	164	717	35661	2557	3743	665	36214	2228

^a^data from *Salmonella *Annual Summary for clinical *Salmonella *isolates from nonhuman and human sources reported to the Disease Control and Prevention (CDC) and the USDA National Veterinary Services Laboratory (NSVL), USA.

^b^data from Annual Report and Accounts 2008/2009 of Veterinary Laboratory Agency, Department of Environment, Food and Rural Affairs, United Kingdom.

^c^data from the Disease Control and Prevention (CDC), Taiwan.

## Discussion

As one of main pathogen to cause foodborne diseases, *Salmonella *has been frequently reported among different animal sources, especially more divergent *Salmonella *serovars found in chickens [34]. With the limited serovars in 164 chicken isolates, serogroups C2, D, E and G were restricted in one county and serogroup B and C1 were found in all three counties (Table 2), suggesting possibly that serogroup B and C1 isolates may be more adapted to chicken. In human isolates, we found that the serovar number in each serogroup were not associated positively with the serogroup prevalence, such as highest serovar number in low prevalent serogroup C1 vs lower serovar number in high prevalent serogroup B and serogroup D (Table 4). These results imply that serogroup C1 may occasionally infect human isolates. Further, serovars are determined by flagellins: H1 and H2 antigens encoded by *fliC *and *fljB*. As one of the most important immunogens, flagellin interacts with the toll-like receptor 5 (TLR5) to activate NFκB pathway and proinflammatory genes to regulate innate and adaptive immune system [35-38]. However, aflagellar serovars *S*. Pullorum and *S*. Gallinarum cause more severe infection than flagellar serovars in chicken because of aflagellar *S*. Typhimurium could avoid the TLR5 regulation of IL-1β expression and polymorphonuclear cell infiltration in gut [39]. Such evasion of TLR5 is critical for survival of flagellar bacteria at muscos [40]. [In the present study, we found that i of H1 antigen and lack of H2 antigen were the common antigens for all serogroups in human isolates (Table 4). However, in comparing 13 chicken serovars and 66 human serovars of this study with serovars of chicken and human isolates from UK and USA, only *S*. Enteritidis, *S*. Typhimurium, *S*. Albany, *S*. Derby, *S*. Anatum and *S*. Havana were common in both hosts (Table 5). However, these serovars shares same antigens: g complex; i; and z_4_,z_24 _of H1 antigen and 1 complex and - of H2 antigens (Table 5), implying these antigens may be important for *Salmonella *transmission between chicken and human.

Prevalent serogroups and serovars are related to chicken lines (Table 1)[9,10] and ages [15]. In layer, age-related prevalence was reported earlier [15] and no *Salmonella *was isolated from 1-year-old layers in the present study (Table 1). Such age-associated clearance may be due to stronger antigen-specific T-cell response in older chicken [41] and not related to B-cell response [42]. Age-related serovars were also identified in Taiwan broiler chickens (Table 2). Almost all isolates were *S*. Choleraesuis and non-typable *Salmonella *(possibly monophasic *S*. Choleraesuis) of serogroup C1 in Chick group and *S*. Mons of serogroup B in NHC group (Table 2). As swine-adapted pathogen, *S*. Cholearesuis has seldom reported from chicken. However, *S*. Choleraesuis in 1-day-old chicks may be contaminated from the hatchery, particular from eggshell membrane; in which *S*. Typhimurium, not *S*. Choleraesuis, is main serovar [43]. If highly invasive *S*. Choleraesuis could infect chicks and use the chicken as reservoir, it will lead to a public problem of circulating such high invasive serovar in animals. In broiler, prevalence of *Salmonella *differed between chicken parts (2.36% for legs and 4.25% for breasts of broiler) [19]. Further, prevalent serovars differ between sampling sources e.g. the *S*. Anatum and *S*. Rissen in chicken meat [44] and *S*. Blockley, *S*. Hadar and *S*. Bredeney in the cecal samples (24).

Several methods have been developed to differentiate clinical isolates. In this study, PFGE patterns almost matched serotypes, although *S*. Albany and *S*. Havana appeared multiple genotypes with highly similar banding patterns (Table 2). Therefore, PFGE typing is a useful tool to assist serotyping of *Salmonella *isolates before doing traditional serotypes [2,27]. In contrast to PFGE type, plasmid analysis is the most convenient method for subtyping [15,45]. In this study, plasmid variations were more diverse than genomic variations; however, *S*. Albany and *S*. Havana with highly genomic variations lacked plasmid (Table 2). These results may imply that recent evolution of *Salmonella *might be mainly through plasmid acquisition to introduce beneficial genes for host serovar to survival.

Antimicrobial susceptibility of *Salmonella *can be used to monitor drug abuse in different regions (Table 2) [46] and animal sources [44,47]. Early study reported that *Salmonella *from chicken, not from human, pig and cattle, was less resistance to A, C, and Sxt [47]. Nevertheless, resistance to T was frequently found in chicken isolates [48]. Since discovery of ACSSuT-resistant region in SGI of *S*. Typhimurium DT104 [49], variations within SGI and complex integron In*104 *change the antimicrobial resistance [50]. In this study, our chicken isolates were highly resistant to antimicrobials A, C, S, Sxt, T and Ub (Table 3). These results imply that *S*. Albany, *S*. Anatum, *S*. Grmpian, *S*. Hissar, *S*. Kubacha, *S*. Mons, and *S*. Typhimurium with resistance types from H to M may be derived from misuse of antimicrobials or due to presence of SGI and/or integron [51]. Mechanism to develop En and Ci resistance is due to mutation in quinolone-resistance determining region or expression of efflux pump [52]. Earlier, fluoroquinolone-resistant *Salmonella *was seldom reported in poultry's isolates worldwide [10,44,47,48]. Until recently, resistance to similar fluoroquinolones: En and Ci has been reported from chicken in Spain [16]. In contrast to same prevalence of resistance to En and Ci in swine and human isolates [32], we found that resistance rate to En was higher than that of Ci (Table 2). However, En and Ci resistant isolates were only found in few serovars of serogroups B and C1 and mainly in Pintung area (Table 3). These results indicate that possibly En was misuse in Pintung county to induce resistance in prevalent serovars.

## Conclusion

13 chicken serovars were identified and differed in drug resistance and prevalence associated with chicken lines, ages and regions. Five serovars were common between these chicken serovars and 66 human serovars

## Abbreviations

A: ampicillin; BHIA: brain heart infusion agar; C: chloramphenicol; CDC: Center for Disease Control; Ci: ciprofloxacin; CIT: Simmons' citrate agar; Cro: ceftriaxone; CZ: cefazolin; En: enrofloxacin; GN: gram-negative broth; LIA: lysine iron agar; MDR: multi-drug resistance; MHA: Mueller-Hinton agar; MHB: Mueller-Hinton broth; MIO: mobility-indole-ornithine agar; NHC: native hybrid chicken; ORN: Moller's ornithine decarboxylase medium; PFGE: pulsed-field gel electrophoresis; S: streptomycin; SIM: sulfide-indole-kmotility medium; Sxt: sulfamethoxazole-trimethoprium; T: tetracycline; TSI: triple sugar iron agar; Ub: flumequine; URE: Christensen's urea agar; VP: Voges-Proskauer medium; XLD: xylose lysine deoxycholate agar.

## Authors' contributions

CC designed, instructed and supervised most aspects of this project. LHC, CYL and CYY collected samples and data analysis of chicken isolates. LHC and CMY did laboratory work and data analysis. JML and SWC performed the experiments and data analysis. CHC and CSC assisted in the design of the study and data analysis of human isolates. CLC, CYY, and CCH gave useful comments and critically read the manuscript. YMH and CPW assisted in animal sampling, data analysis and edited the manuscript. All authors read and approved the final manuscript.

## Authors' information

L-HC and C-YL are officials of Animal Disease Control Center ChiaYi County, Taiwan; C-HC is professor of Department of Pediatrics, Chang Gung Children's Hospital and Chang Gung University College of Medicine, Taoyuan, Taiwan; Y-MH and C-PW are professors of Department of Animal Science, National Chiayi University, Chiayi, Taiwan; C-MY was master graduate student of Department of Animal Science, National Chiayi University, Chiayi, Taiwan; C-SC is Chief Investigator of The Central Region Laboratory, Center of Research and Diagnostics, Centers for Disease Control, Taichung, Taiwan; C-YY is professor of Department of Veterinary Medicine, National Chiayi University, Chiayi, Taiwan; C-CC is associate professor of Graduate Institute of Veterinary Public Health, School of Veterinary Medicine, National Chung Hsing University, Taichung, Taiwan; CC is the chairman of Department of Microbiology and Immunology, National Chiayi University, Chiayi, Taiwan.

## Supplementary Material

Additional file 1 **Table S1. Association of antibiograms with serogroups among three counties**. Antibiograms differed among three counties and serogroups. Click here for file

Additional file 2 **Table S2. Plasmid profiles of serovars in each serogroup**. Plasmid profiles determined by size and number was associated with serotypes. Click here for file

Additional file 3 **Figure S1. Representative plasmid profiles of *Salmonella *isolates collected from chickens**. Plasmid size and number of each representative plasmid profile was determined by Kado-Liu method and standard plasmid size of 50 kb and 90 kb plasmid of OU7526. Click here for file
