# Supplementary material for: Characterization of 13 multi-drug resistant Salmonella serovars from different broiler chickens associated with those of human isolates
Source: BMC Microbiol. 2010 Mar 23;10:86. doi: 10.1186/1471-2180-10-86 (PMC2859872; doi:10.1186/1471-2180-10-86)
Supplement: Additional file 1 — Table S1. Association of antibiograms with serogroups among three counties. Antibiograms differed among three counties and serogroups. [file 1471-2180-10-86-S1.PDF]

Table S1. Association of antibiogram with serogroups among three counties

County	Chiayi			Tainan			Pintung			
Serogroup / Antibiogram	B	C1	G	B	C1	D	B	C1	C2	E
A		77	2							
B						8				
C	1									
D							1			
E							1		1	
F						10				
G					2		1			
H										2
I	12	1								
J	4			11					10	3
K	1			2						
L				1						
M							4	8		
Total	18	78	2	14	2	18	7	8	11	5
