# Supplementary material for: Characterization of 13 multi-drug resistant Salmonella serovars from different broiler chickens associated with those of human isolates
Source: BMC Microbiol. 2010 Mar 23;10:86. doi: 10.1186/1471-2180-10-86 (PMC2859872; doi:10.1186/1471-2180-10-86)
Supplement: Additional file 2 — Table S2. Plasmid profiles of serovars in each serogroup. Plasmid profiles determined by size and number was associated with serotypes. [file 1471-2180-10-86-S2.PDF]

Table S2. Plasmid profiles of serovars in each serogroup

Serogroup	Plasmid type	Plasmid number and size (kb)				Total plasmid	Serovars
		< 20	20 - < 50	50 - 90	> 90		
B	1			1		1	Kubacha, Mons
	1a	4		1		5	Mons
	1b		1	1		2	Mons
	1c	4	1	1		6	Mons
	1d	1		1		2	Mons
	2			1	1	2	Mons
	2a	4			1	5	Mons
	2b	2			1	3	Mons
	3				2	2	Mons
	3a	1	1		2	4	Typhimurium
	4			2		2	Typhimurium var Copenhagen
	4a			2	1	3	Kubacha
	5	4			0	4	Derby
	6					0	Typhimurium
C1	1		1		1	2	Choleraesuis, NT
	1a		1		1	2	Grampian
	2	1				1	NT
	3			2		2	Grampian
	4	2				2	Hissar
	5					0	Choleraesuis, Redba
C2	1					0	Blockley, Albany
D	1			1		1	Enteritidis
	2			1	1	2	Enteritidis
	3					0	Enteritidis
E	1			1	1	2	Anatum
	2		1	1	2	4	Anatum
G	1					0	Havana

NT: non-typable
