# Supplementary material for: Characterization of 13 multi-drug resistant Salmonella serovars from different broiler chickens associated with those of human isolates
Source: BMC Microbiol. 2010 Mar 23;10:86. doi: 10.1186/1471-2180-10-86 (PMC2859872; doi:10.1186/1471-2180-10-86)
Supplement: Additional file 3 — Figure S1. Representative plasmid profiles of Salmonella isolates collected from chickens. Plasmid size and number of each representative plasmid profile was determined by Kado-Liu method and standard plasmid size of 50 kb and 90 kb plasmid of OU7526. [file 1471-2180-10-86-S3.PDF]

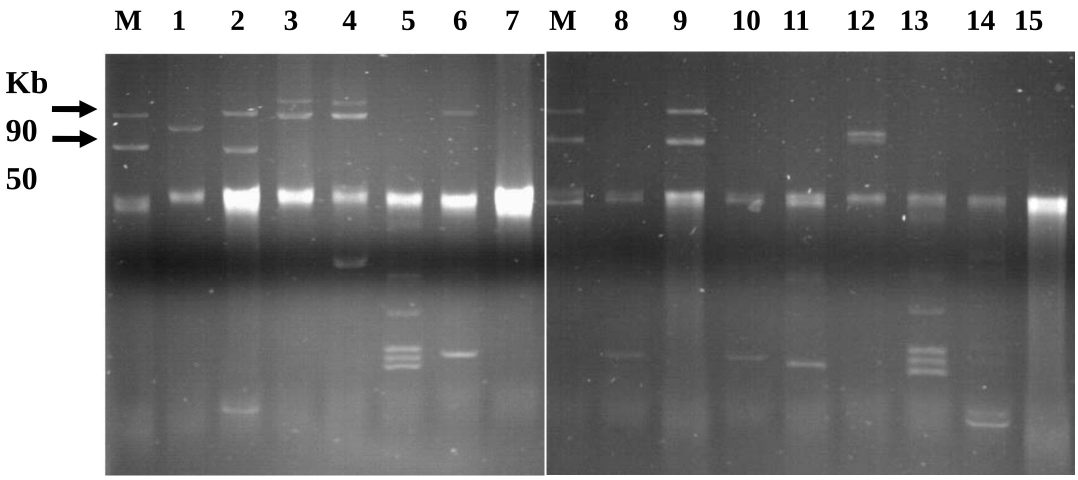

Figure S1. Representative plasmid profiles of *Salmonella* isolates collected from chickens. M: *S.* Choleraesuis isolate OU7527 harbors 90-kb and 50-kb plasmids used as plasmid size marker. Lane 1: SW2 of S. Mons, lane 2: TN1 of S. Mons, lane 3: TN31 of S. Mons, lane 4: P10 of *S.* Typhimurium, lane 5: P1 of *S.* Derby, lane 6: SP1 of S. Mons, lane 7: P19 *S.* Albany, lane 8: SC1 of non-typable serogroup C1 *Salmonella*, lane 9: SC7 of S. Choleraesuis, lane 10: SC70 of non-typable serogroup C1 *Salmonella*, lane 11: SC83 of non-typable serogroup C1 *Salmonella*, lane 12: TN32 of *S.* Grampian, lane 13: P13 of *S.* Hissar., lane 14: SW1 of *S.* Grampian, and lane 15: SC55 of *S.* Choleraesuis . Plasmid DNAs were separated by 0.6% agarose at 50 V for 1.5 hrs.
